# Prognostic value of metabolic parameters measured by pretreatment dual-time-point ^18^F-fluorodeoxyglucose positron emission tomography/computed tomography in patients with intrahepatic or perihilar cholangiocarcinoma: A STROBE study

**DOI:** 10.1097/MD.0000000000026015

**Published:** 2021-05-28

**Authors:** Jae Pil Hwang, Jong Ho Moon, Hee Kyung Kim, Min Hee Lee, Chae Hong Lim, Soo Bin Park, Joon-Kee Yoon, Jung Mi Park

**Affiliations:** a Departments of Nuclear Medicine; b Departments of Internal Medicine; c Departments of Pathology; d Departments of Radiology, Soonchunhyang University Hospital Bucheon, Bucheon; e Departments of Nuclear Medicine, Soonchunhyang University Hospital Seoul, Seoul; f Department of Nuclear Medicine, Ajou University Medical Center, Suwon, Republic of Korea.

**Keywords:** dual-time point positron emission tomography/computed tomography, intrahepatic cholangiocarcinoma, maximum standardized uptake value, perihilar cholangiocarcinoma

## Abstract

The purpose of this study was to determine the glucose metabolism at delay phase measured by pretreatment dual-time-point ^18^F-fluorodeoxyglucose (^18^F-FDG) positron emission tomography (PET)/ computed tomography (CT) provides prognostic information independent of well-known prognostic factors in patients with intrahepatic or perihilar cholangiocarcinoma (ICC or PCC).

From July 2012 to December 2017, 55 patients (men 27, women 28, mean age 68 ± 11 years) with pathologically proven ICC or PCC were enrolled in this retrospective study. The dual-time-point ^18^F-FDG PET/CT as part of a staging workup was performed in all patients. The patient's data includes age, sex, serum CA19-9, presence of LN or distant metastasis, early SUVmax (early maximum standardized uptake value [eSUV]), delay SUVmax (delay maximum standardized uptake value [dSUV]), retention index of SUVmax (percent change of maximum standardized uptake values [ΔSUV]), neutrophil to lymphocyte ratio (NLR) and histopathology including pCEA, p53, Ki-67 index. The analysis of the relationship between metabolic parameters and survival was done using the Kaplan–Meier curve and Cox proportional hazards regression model.

Median survival for all patients was 357 days. Median early and delay SUVmax was 5.2 (range: 2.0–21.4) and 6.5 (range 2.7–24.5), respectively. The overall survival was found to be significantly related to eSUV, dSUV, ΔSUV, age, serum CA19-9 and NLR in univariate analysis. In multivariate analysis, dSUV (*P* = .014, 95%CI; 1.30–10.7, HR 3.74) and ΔSUVmax (*P* = .037, 95%CI; 1.05–6.12, HR 2.5) were independent factors of overall survival. Kaplan–Meier curve analysis clearly showed the significant difference of overall survival between 2 groups (high eSUV, low eSUV + high ΔSUV vs low eSUV and ΔSUV, *P* < .001) among the comparisons of the SUV parameters on FDG PET. In the receiver operating characteristic analysis using combinations of the SUV parameters, the 2 groups [eSUV + ΔSUV (*P* = .0001, area under the curve [AUC] 0.68) and dSUV + ΔSUV (*P* = .0002, AUC 0.71)] showed significantly larger AUC than the other groups applying eSUV or dSUV alone (AUC 0.61 and AUC 0.68).

dSUV and ΔSUV on pretreatment dual-time-point ^18^F-FDG PET/CT can be useful parameters in the prediction of survival in patients with ICC or PCC.

## 1 Introduction

Cholangiocarcinoma is a rare malignancy that arises from bile duct epithelium and is usually classified into intrahepatic, perihilar (Klatskin tumor) and extrahepatic bile duct. A major proportion of tumors, 60% to 70% are perihilar, 20% to 30% are located distal bile duct and the intrahepatic portion accounts for 5% to 10%.^[1]^ Because intrahepatic cholangiocarcinoma (ICC) and perihilar cholangiocarcinoma (PCC) are generally asymptomatic and are diagnosed when the disease has already advanced progression, late diagnosis can miss an opportunity of proper treatment decision such as surgical resection and/or chemotherapy. Besides, the recurrence rate and chemoresistance of this disease are very high; thus, it is important to predict the prognosis for the decision regarding whether treatment should be continued or changed.^[2]^

Recently, the diagnostic value of ^18^F-fluorodeoxyglucose (^18^F-FDG) positron emission tomography (PET)/CT such as staging, detection of recurrent tumor, decision-making of a treatment plan, and prognostic value has been well established in bile duct malignancies, in addition to a diagnostic value of conventional diagnostic imaging modalities such as endoscopic retrograde cholangiopancreatography, ultrasonography, computed tomography (CT), and magnetic resonance imaging.^[3–7]^

Recent studies have used dual-time-point ^18^F-FDG PET/CT to better diagnostic or prognostic accuracy for various malignant disease, because of the more prominent ^18^F-FDG uptake in malignant conditions, whereas with less ^18^F-FDG uptake in benign or non-tumorous conditions on a delayed scan.^[8–13]^ But few studies have applied this technique for assessment of malignant potential in patients with extrahepatic bile duct cancer or biliary strictures.^[14–16]^ Because dual-time-point imaging technique has been known to overcome partial volume effect and to emerge as prominent small lesion, this technique might be helpful to bile duct cancers with small and narrow structure.^[17,18]^ And SUVmax change over time such as percent change of maximum standardized uptake values (ΔSUV) measured by dual-time-point imaging has advantage as high predictive value. And, to our knowledge, the prognostic value of dual-time-point ^18^F-FDG PET/CT scan inpatients with ICC and PCC have not yet been evaluated.

This retrospective study was designed to assess whether delay metabolic parameter achieved by dual-time-point ^18^F-FDG PET/CT provides prognostic information independent of well-known prognostic factors in patients with ICC and PCC.

## 2 Methods

### 2.1 Patient characteristics

Between July 2012 to December 2017, 55 consecutive patients with pathologically proven ICC or PCC who underwent pretreatment dual-time point ^18^F-FDG PET/CT were retrospectively reviewed. Pretreatment conventional imaging modalities included ultrasonography, CT, and magnetic resonance imaging for characterization of tumor and initial staging workup. Patient data were recorded as follows: age, sex, serum CA19–9, presence of LN or distant metastasis based on the radiologic findings, early SUVmax (early maximum standardized uptake value [eSUV]), delay SUVmax (delay maximum standardized uptake value [dSUV]), retention index of SUVmax (ΔSUV), neutrophil to lymphocyte ratio (NLR) and histopathology of primary tumor including pCEA, p53, Ki-67 index. NLR was calculated by dividing the absolute neutrophil count by the absolute lymphocyte count, which was one of independent prognostic factors by the previous studies.^[19–21]^ The institutional review board of Soonchunhyang University Hospital Bucheon approved this retrospective study, and the requirement to obtain informed consent was waived because of retrospective design of the study.

### 2.2 Study design setting for patient sample size

The estimation was done in the way as followed; predicting hazard ratio 4.28 using SUVmax 7.3 of cut off in ICC or PCC with overall survival analysis was referred and the sample sizes in 2-group survival analysis were estimated by the web-based calculator program at UCSF (https://sample-size.net/sample-size-survival-analysis).^[22]^

In the assumption of the following values such as σ =0.05, estimating power 90%, the subject proportion of the exposed group = 0.6, relative hazard (exposed group/unexposed group) = 4.0, total death events estimated 23. And under the assumption conditions of 0.5 baseline event rate, 1 years median survival time for unexposed group, and 50% of censoring rate, 3 years of follow up duration, the estimation sample sizes were 34 for the total group, 20 for the exposed group and 14 for the unexposed group, respectively. We proposed at least 34 patients as the total sample size and at least 23 death events of this study for 2-group survival analysis.

### 2.3 ^18^F-FDG PET/CT scan

All PET/CT scans were performed using hybrid PET/CT systems (Biograph 128mCT, Siemens Medical Solutions, Knoxville, TN). Patients were educated too fast for at least 6 hours and the peripheral blood glucose level was <120 mg/dL. Patients received an intravenous injection of 370 to 555 MBq at 1 hour prior to imaging and no intravenous contrast agent was administered. The first scan was performed using 6 to 8 bed positions and the acquisition time was 2 to 3 minutes per bed position. The CT scan was performed at 100 kVp, 50 mA, and 5 mm slice thickness. Approximately 2 hours after ^18^F-FDG injection, the delay phase PET/CT images over the center of malignancy on the early images were acquired and conventional imaging studies. The PET images were reconstructed using the CT based standard ordered subset expectation maximization algorithm.

### 2.4 Image analysis

All PET/CT images were evaluated by consensus between 2 experienced nuclear medicine physicians on an interactive computer display using fusion software (Syngo; Siemens Medical Solutions). For semi-quantitative analysis, the maximum standardized uptake value (SUVmax) was measured by placing region of interest over the maximal hypermetabolic area suspected to be a malignant focus from the eSUV and dSUV were obtained at 1-hour and 2-hour images after I.V. injection, respectively. The SUV was calculated using the following equation: decay-corrected activity [kBq]/ml of tissue volume/injected ^18^F-FDG activity [kBq]/g of body mass. A receiver operating characteristic (ROC) curve analysis was used to determine the optimal cut-off value of the dSUV.

The ΔSUV was calculated as follows:

ΔSUV(%)=[(dSUV−eSUV)/eSUV]×100%

### 2.5 Statistical analysis

The statistical analysis was performed using Medcalc software v. 11.3 (Medcalc Software, Ostend, Belgium). Survival time was defined as the time from pre-treatment ^18^F-FDG PET /CT scan to the date of the detection of death or to the date of the last follow up visit at our hospital. The mean duration of clinical follow-up was 36.7 ± 3.7 month (median, 33 months; range, 6–82 month). Overall survival curve was analyzed by the Kaplan–Meier method with log-rank test, and *P* values were less than .05 as statistically significant. Variables with *P* < .05 in the univariate analysis of factors affecting survival were included in a subsequent multivariate analysis using Cox proportional hazard model. The ROC curve analysis was used to determine the optimal cut off values of clinical data and metabolic parameters of ^18^F-FDG PET/CT in predicting survival or death. The areas under the curve (AUC) were calculated and the optimal cut off values of each metabolic parameter were evaluated. To compare clinicopathologic factors and metabolic parameters between patients in the high and low SUVmax groups, Mann–Whitney *U* test, Fisher exact test, and the chi-squared test were applied additionally.

## 3 Results

### 3.1 Patient characteristics

A total of 55 patients were enrolled in our study and the patients’ characteristics are detailed in Table 1. The mean age of the patients was 68.5 ± 11.4years (range, 47–94 years; 27 men and 28 women). Median survival for all 55 study subjects was 357 days and median early and delay SUVmax was 5.2 (range: 2.0–21.4) and 6.5 (2.7–24.5). Positive cases for LN metastasis were 32 (58%) and distant metastasis were 8 (14%). For median laboratory values, serum CA19–9 and NLR, were 132 U/mL and 3.4. The classifications of primary tumors were 24 ICC (44%) and 28 PCC (56%). All patients were undergoing surgery (n = 14) or chemotherapy (n = 41).

**Table 1:** Characteristics of patients.

Characteristics	Number of patients (n = 55) [(n (%)]
Age (mean ± SD) (yr)	68.5 ± 11.4
Sex	
Male	27 (49%)
Female	28 (51%)
Classification	
ICC	24 (44%)
PCC	31 (56%)
LN metastasis on CT/MR	
Yes	32 (58%)
No	23 (42%)
Distant metastasis on CT/MR	
Yes	8 (14%)
No	47 (86%)
serum CA19-9 (U/mL)	
≥ 324	35 (64%)
< 324	20 (34%)
Neutrophil to lymphocyte ratio (NLR)	
≥ 2.9	26 (47%)
< 2.9	29 (53%)
Histopathologic findings	
CEA expression	
Yes	35 (64%)
No	20 (35%)
p53 expression	
Yes	26 (47%)
No	29 (53%)
Treatment	
Surgery	14 (26%)
Chemotherapy	41 (74%)
Survival (median, mo)	11.9 (6.0–55.2)

### 3.2 Patient characteristics between high and low groups divided by dSUV

The correlations between high and low dSUV groups with clinicopathological and metabolic parameters are presented in Table 2. We divided subgroups according to the cut-off value of dSUV 7.3, high dSUV groups showed higher mean SUVmax than that of low dSUV group (mean SUVmax 10.1 ± 4.9 vs 4.3 ± 1.3, *P* = .04) and shorter survival days (331.9 days vs 558.3 days, *P* = .01). Distant metastasis and NLR showed significant differences of distribution of patients according to dSUV (*P* = .002 and *P* = .034, respectively). LN metastasis showed a borderline significant tendency to dSUV (*P* = .05). ICC showed a similar distribution rate of low and high dSUV, on the contrary, PCC tended to have lower dSUV. (*P* = .01)

**Table 2:** Patient characteristics for groups with delay SUVmax.

	Low dSUVmax (n = 35)	High dSUVmax (n = 20)	*P* value
dSUVmax (mean ± SD)	4.3 ± 1.3	10.1 ± 4.9	
Survival (median) (mo)	18.6 ± 14.4	11.1 ± 12.7	.01
Age (mean ± SD) (yr)	66.0 ± 11.2	71.9 ± 10.0	.14
Sex			
Male	17	10	.91
Female	18	10	
Classification			
ICC	13	11	.01
PCC	22	9	
LN metastasis on CT/MR			
Yes	17	15	.05
No	18	5	
Distant metastasis on CT/ MR			
Yes	1	7	.002
No	34	13	
serum CA19-9 (U/mL)			
≥ 324	24	11	.32
< 324	11	9	
Neutrophil to lymphocyte ratio (NLR)			
≥ 2.9	18	11	.03
< 2.9	17	9	
Histopathology			
CEA expression	22	13	.87
Yes	13	7	
No			
p53 expression			
Yes	16	10	.76
No	19	10	.37
Treatment			
Surgery	10	4	
Chemotherapy	25	16	

### 3.3 Comparison of survival curves

**Figure 1:**
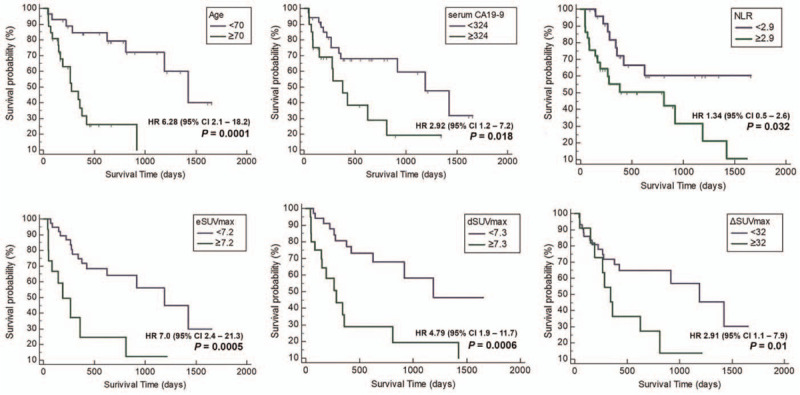
Kaplan–Meier (K–M) curve analysis for clinical and metabolic factors for overall survival.

**Figure 2:**
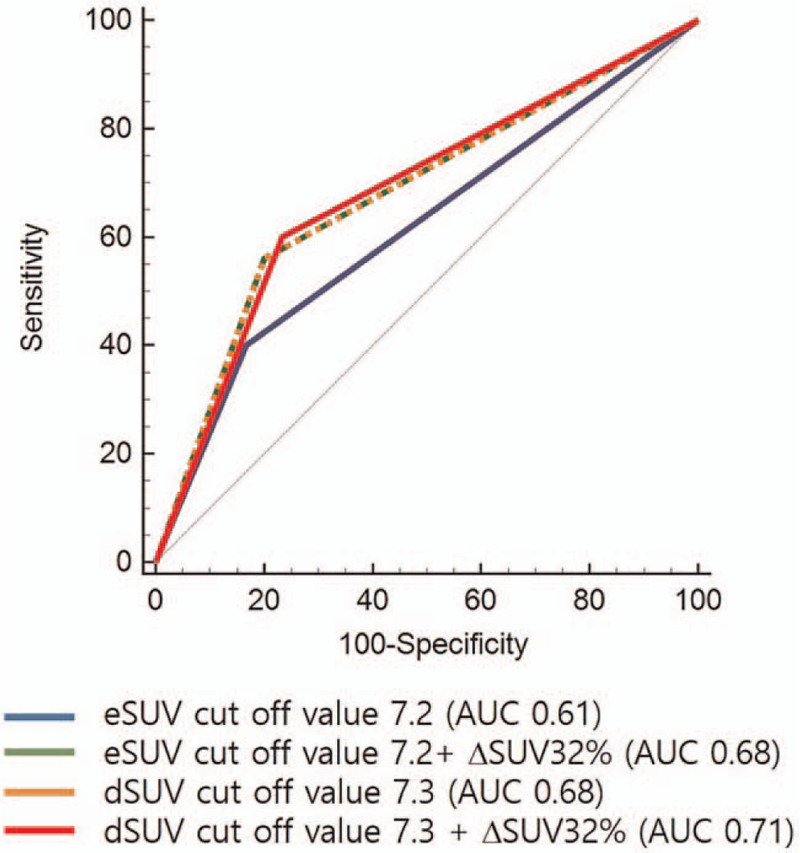
ROC curve analysis for metabolic parameters with combination of eSUV, dSUV, and ΔSUV.

Univariate analysis revealed that the survival was found to be significantly related to eSUV 7.2 (cut-off value 7.2, *P* < .001), dSUV 7.3 (cut-off value 7.3, *P* < .001), ΔSUV 32% (cut-off value 32%, *P* = .01), age (*P* < .001), NLR 2.9 (cut-off value 2.9, *P* = .032) and serum CA19-9 324 (cut-off value 324 U/mL, *P* = .018) (Fig. 1). Variables for which *P* < .05 in the univariate analysis were included in the multivariate analysis, dSUV (*P* = .014, 95%CI: 1.30–10.7, HR 3.74), ΔSUV (*P* = .037, 95%CI: 1.05–6.12, HR 2.5), NLR (*P* = .041, 95%CI: 1.03–6.02, HR 2.5), serum CA19-9 (*P* = .014, 95%CI: 1.23–7.09, HR 2.9) and age (*P* = .007, 95%CI: 2.15–17.30, HR 6.1) were independent factors of overall survival (Table 3). In addition, AUC graphs were analyzed to predict overall survival in using of each PET parameter such as eSUV, Dsuv, and ΔSUVmax. Predicting power of each PET parameters were high in the order of dSUV 7.3% to ΔSUV 32% (AUC 0.71), dSUV 7.3 alone (AUC 0.68), eSUV 7.2 toΔSUV 32% (AUC 0.68), and eSUV 7.2 alone (AUC 0.61) (Fig. 2).

**Table 3:** Univariate and multivariate analysis for overall survival.

	Univariate analysis			Multivariate analysis		
Variable	No.	95% CI	*P* value	HR	95% CI	*P* value
eSUVmax			.0005	2.11	0.71–6.61	.169
dSUVmax			.0006	3.74	1.30–10.76	.014
ΔSUVmax			.01	2.50	1.05–6.12	.037
Age (yr old)						
≥ 70	29	2.16–18.23	.0001	6.11	2.15–17.30	.007
< 70	26					
Sex						
Male	27	0.58–3.17	.87			
Female	28					
Classification						
ICC	24	0.19–1.49	.81			
PCC	31					
LN metastasis						
Yes	32	0.35–2.23	.141			
No	23					
Distant metastasis						
Yes	8	0.29–4.03	.367			
No	47					
Serum CA19-9						
≥ 324 U/mL	20	1.25–7.28	.018	2.96	1.23–7.09	.014
< 324 U/mL	35					
NLR						
≥ 2.9	26	0.49–2.61	.032	2.49	1.03–6.02	.041
< 2.9	29					
CEA expression						
Yes	35	0.27–1.46	.754			
No	21					
p53 expression						
Yes	26	0.75–3.92	.221			
No	29					
Combination of SUVmax indices						
eSUV and dSUV			0.0001			.003
eSUV and ΔSUV			0.0001			.002
dSUV and ΔSUV			0.0068			.0002

### 3.4 Comparisons of survival according to the combination of early and delay SUVmax

**Figure 3:**
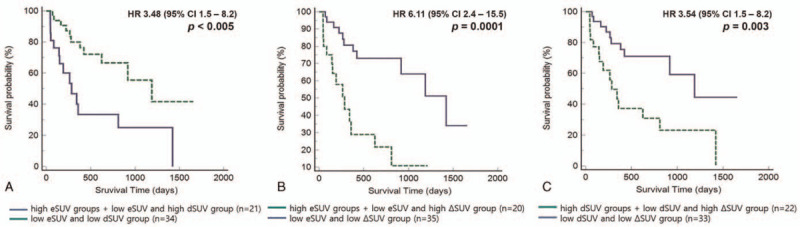
Kaplan–Meier (K–M) analysis for early and delay metabolic parameters for overall survival. (A) early SUVmax and delay SUVmax, (B) early SUVmax and ΔSUVmax, (C) delay SUVmax and ΔSUVmax.

For predicting overall survival, the patients were divided into 2 groups according to the combination of both eSUV 7.2 and dSUV 7.3: group (N = 21, 14 high eSUV-high dSUV; 2 high eSUV-low dSUV; 5 low eSUV-high dSUV) and group (34 low eSUV and low dSUV). In Figure 3A, there was a significant differences of overall survival curves between 2 groups (median survival duration 190 days vs 1672 days, *P* < .005). In addition, the analysis for predicting overall survival was performed by dividing the patients into 2 groups according to the combination of both eSUV 7.2 and ΔSUV 32%: the group (N = 20, 6 high eSUV-high ΔSUV; 9 high eSUV-low ΔSUV; 5 low eSUV-high ΔSUV) and the group (35 low eSUV and low ΔSUV) (Fig. 3B). There was a significant differences of overall survival curves between 2 groups (median survival duration 177 days vs 453 days, *P* < .001). In the results of the combination of both dSUV 7.3 and ΔSUV 32% was perfomed for predicting overall survival: the group (N = 22, 9 high dSUV-high ΔSUV; 11 high dSUV-low ΔSUV; 2 low dSUV-high ΔSUV) and the group (33 low dSUV and low ΔSUV) (Fig. 3C). There was a significant differences of overall survival curves between 2 groups (*P* = .003).

## 4 Discussion

Our study evaluated the relationship between metabolic parameter of the primary tumor and prognosis in patients with ICC and PCC. Also this study demonstrated that the dSUV and ΔSUV of the primary tumor were independent prognostic factors. dSUV, ΔSUV, age, NLR and serum CA19-9 level were significantly correlated with overall survival in univariate analysis. In the ROC analysis, combination of dSUV and ΔSUV showed a significantly higher predicting value of overall survival than the other subgroups. (AUC 0.71) In addition, the groups including high dSUV or ΔSUV showed a significant poor overall survival than the groups including low dSUV or ΔSUV. Multivariate analysis revealed that overall survival was significantly influenced by dSUV and ΔSUV. The hazard ratio for the higher dSUV scores was 3.7 times that of the lower dSUV groups and was independent of other prognostic factors. According to our results, dSUV and ΔSUV calculated from dual-time-point ^18^F-FDG PET/CT are strong independent prognostic parameters of ICC and PCC, allowing accurate identification and detailed risk stratification of patients who will benefit from intensive and proper treatment.

Previous studies have investigated the prognostic value of baseline SUVmax measured by ^18^F-FDG PET/CT in patients with biliary tract cancer. Tomoaki et al reported that high tumor baseline SUVmax represents poor prognosis in multivariate analysis in patients with ICC.^[5]^ Sabate et al reported that higher tumor-to-liver ratio were significantly related with shorter overall survival in ICC or extrahepatic cholangiocarcinoma (ECC).^[3]^ Angela et al investigated that baseline high SUVmax was associated with worse survival through systemic review and meta-analysis in patients with biliary tract cancer.^[23]^ The present study showed that there was no significant prognostic difference of eSUV in multivariate analysis, although it was significant correlated overall survival in univariate analysis. We measured metabolic parameters of the primary tumor excluding any metastatic lesion, which may affect different survival outcome. In addition, different tumor categories may be also attributable for different result.

In a previous study, Park et al reported that SUVmax from both early and delay PET/CT scans is useful parameters in the classification of extrahepatic biliary malignancy from benign disease, but there was no added benefit of delay PET/CT inpatients suspicious for extrahepatic cholangiocarcinoma. They focused on diagnostic performance distinguish malignant from benign disease using delay metabolic imaging parameters (SUVmax2 and %ΔSUVmax) in patients with extrahepatic cholangiocarcinoma. On the other hand, we focused on the prediction of overall survival using early and delay SUVmax, and we suggested dual-time-point metabolic parameters (dSUV and ΔSUV) could be useful as a prognostic value for overall survival in multivariate analysis.

Other previous study, Lee et al reported that high SUVmax, SUVpeak, SUVmean, SUVgluc and TLGgluc showed significantly shorter overall survival in univariate analysis and operability was independent prognostic factor in multivariate analysis in patients with ICC. Compared to this previous study using single time point PET/CT, we suggested a prognostic value of dual-time-point metabolic parameters (dSUV and ΔSUV) for overall survival in multivariate analysis.

In another study using dual-time-point metabolic parameter in another malignancy, Tsuda et al reported that dual time PET/CT parameters can be a useful method for predicting relapse in patients with breast cancer and that SUVmax1 and %ΔSUVmax were able to identify the worse prognostic subgroups more accurately than SUVmax1 alone.^[24]^ Our study showed the similar results that delay SUVmax and ΔSUVmax are independent prognostic factors of overall survival, and more detailed risk stratification is possible combining dSUV and ΔSUV parameters to those of previous study.

Age and serum CA19-9 are well known clinical prognostic factors of bile duct cancer.^[25–29]^ Most of patients with bile duct cancer occur in over the age of 50 and the risk of incidence increases with age. Our study revealed similar result of significant prognostic factor of cholangiocarcinoma in multivariate analysis as well as metabolic prognostic parameters measured by ^18^F-FDG PET/CT. In addition, LN metastasis, distant metastasis and tumor classification (ICC or PCC) are also well-known prognostic factors. But, the prognostic difference between those factors and overall survival was not statistically significant in multivariate analysis, although these factors showed a significantly difference in patients from the high dSUV group than in those from low dSUV group in our study. This is maybe to be influenced by small number of patients, heterogenous tumor category or determination dependent on the radiologic findings.

Previous studies about change of metabolic activity over time on PET/CT scan reported that ^18^F-FDG uptake of malignant lesion continued to increase until approximately 4 to 5 hours after injection, but the uptake of benign lesion decreased 30 minutes after the injection. Furthermore, the %ΔSUVmax was correlated with the grade of malignancy in variable cancers such as lung cancer and/or lymphoma.^[30–33]^ Although the usefulness of %ΔSUVmax was generally considered acceptable, few reports have been published on its relationship with the prognosis of breast cancer.

Many hospitals and institutions have a single time point ^18^F-FDG PET/CT protocol that starts acquiring images at 60 minutes after ^18^F-FDG administration and the SUVmax is obtained at that time. Time course of FDG accumulation in malignant and benign conditions differs along the period of time. Background blood pool activity decreases exponentially soon after the administration of radiotracer. Malignant cells continue to show increasing levels of FDG uptake over time for about 60 minutes, but benign cells such as inflammatory condition reveal a decline pattern due to characterization of FDG-6-phsphate metabolism. The contrast between target tissues (cancer or inflammation) increases substantially over time and the target lesion becomes more distinct.^[34]^ In another study, Alavi et al reported that dual-time-point imaging have been used to overcome the non-specificity of the FDG due to decrease in the background activity in delay time point leads to enhanced lesion detection of various clinical settings. In addition to the dual-time-point imaging has been shown to be helpful in characterization of malignant and benign diseases and prognostication of patients with variable cancers.^[35]^

Cholangiocarcinoma was divided into intrahepatic, perihilar and extrahepatic cholangiocarinoma, they are maybe different in the treatment plan and prognosis according to the cancer types. Perihilar cholangiocarcinoma describes tumors located between the secondary branches of the right and left hepatic ducts and the common hepatic duct proximal to the cystic duct origin. Klatskin tumor classification subtype III and IV describes right or left hepatic duct and both hepatic ducts involvement. The current study includes ICC or PCC excluding the ECC for several reasons as follows: First, PCC could be accompanied by intrahepatic lesion along the tumor infiltration. Second, ICC or PCC have a feature of low operability compared with ECC. Third, a small number of patients of each ICC or PCC were included in our study.

There are several limitations in the current study. First, our study includes heterogenous tumor categories due to relatively small number of patients, maybe it had an effect on the results. Further studies including larger number of patients with increased homogeneity of tumor. Second, it was a retrospective and single center study, these issues may have increased the risk of selection bias.

Some controversy over scan time of dual-time-point PET/CT still remained. Because a standard protocol for dual-time-point imaging has not yet been established, we plan to perform further study with larger number of patients and homogenous tumor category for determine the optimal time point and metabolic parameter of delay imaging for prognostic or diagnostic evaluation.

## 5 Conclusion

The delay SUV and ΔSUV in dual-time-point ^18^F-FDG PET/CT may be significant prognostic factors in patients with ICC and PCC. Dual-time point ^18^F-FDG PET/CT imaging could be a useful tool to provide prognostic information for these patients.

## Author contributions

**Conceptualization:** Jae Pil Hwang, Soo Bin Park, Jung Mi Park.

**Investigation:** Jae Pil Hwang, Jong Ho Moon, Hee Kyung Kim, Min Hee Lee, Chae Hong Lim.

**Methodology:** Jong Ho Moon, Hee Kyung Kim, Min Hee Lee, Chae Hong Lim, Soo Bin Park.

**Writing – original draft:** Jae Pil Hwang, Jung Mi Park.

**Writing – review & editing:** Joon-Kee Yoon, Jung Mi Park.
